# Associations between lower-limb muscle activation and knee flexion in post-stroke individuals: A study on the stance-to-swing phases of gait

**DOI:** 10.1371/journal.pone.0183865

**Published:** 2017-09-08

**Authors:** Wei Wang, Ke Li, Shouwei Yue, Cuiping Yin, Na Wei

**Affiliations:** 1 Laboratory of Motor Control and Rehabilitation, Institute of Biomedical Engineering, School of Control Science and Engineering, Shandong University, Jinan, China; 2 Department of Physical Medicine and Rehabilitation, Qilu Hospital, Shandong University, Jinan, China; 3 Department of Geriatrics, Qilu Hospital, Shandong University, Jinan, China; University of L'Aquila, ITALY

## Abstract

Reduced knee flexion is a leading feature of post-stroke gait, but the causes have not been well understood. The purpose of this study was to investigate the relationship between the knee flexion and the lower-limb muscle activation within the stance-to-swing phases of gait cycle in the post-stroke hemiplegic patients. Ten stroke patients and 10 age- and gender-matched healthy subjects participated in the experiment. The lower-limb kinematic signals and the surface electromyography (sEMG) signals of the left and right rectus femoris (RF), biceps femoris (BF) and lateral gastrocnemius (GS) were recorded during walking. The angle range (AR) of knee flexion, the root mean square (RMS) and the mean frequency (MNF) of sEMG signals were calculated from the terminal stance (TSt) to the initial swing (ISw) phases of gait cycle. Stroke patients showed lower bilateral AR of knee flexion and lower RMS of GS on the paretic side, but higher MNF of RF on the non-paretic side compared with the controls. Within the stroke patients, significant differences were found between their paretic and non-paretic limbs in the AR of knee flexion, as well as in the RMS and MNF of GS (*p* < 0.05). Regression analysis showed that the RMS of BF, MNF of BF and MNF of GS explained 82.1% of variations in AR of knee flexion on paretic side (*r*^*2*^ = 0.821). But the RMS and MNF of all the muscles (including the RF, GS and BF) could explain 65.6% of AR of knee flexion variations on the non-paretic side (*r*^*2*^ = 0.656), and 45.2% of variations for the healthy subjects (*r*^*2*^ = 0.452). The reduced knee flexion during gait was associated with altered magnitude and frequency of muscle contractions and with simplified muscle synergy in the post-stroke hemiplegic patients. Identifying the muscles that are responsible for knee stiffness may facilitate improvement of rehabilitation strategy for post-stroke gait.

## Introduction

Recovery of locomotion ability is a primary goal of post-stroke hemiparesis rehabilitation. The abnormal gait after stroke is characterized by reductions in stride length, movement speed, muscle power and joint range of motion [1–3]. Although all the lower-limb joints could be affected by stroke, the knee would be more vulnerable than the others. The peak knee flexion in the swing phase of gait usually drops off, recognized as the stiff-knee gait, which is a common abnormality associated with stroke. No consensus has been reached regarding the pathophysiology of stiff-knee gait. Over-activation of quadriceps muscle during the swing phase has often been mentioned [4]; while other factors, such as the hip flexor weakness and ankle plantar flexor hyperactivity at the terminal stance phase, may also make a contribution [5]. Identifying the contributors to the stiff knee may facilitate to find more efficient rehabilitation strategy for post-stroke gait.

Among the muscles surrounding the knee, the rectus femoris (RF), gastrocnemius (GS) and biceps femoris (BF) are representative muscles responsible for generating appropriate range of motion. The RF prevents excessive knee flexion, and the BF and GS contribute to the knee flexion during swing of healthy gait. However, roles of these muscles in the post-stroke stiff knee are still controversial. Abnormal excessive activation of RF has been extensively observed in the post-stroke hemiparesis patients and is considered as a primary cause of stiff knee [6]. Some other studies showed that a transfer surgery or a botulinum toxin injection could lower the over-activity of RF, accompanied by the knee flexion improvement during swing [7, 8]. However, Knuppe et al reported that the RF activity during swing phase was not associated with stiff knee after nerve injury [9]. Decreased GS contraction has been found on the pre-swing stage of post-stroke gait, suggesting lowered muscle activation at the initiation of knee flexion during walking [10]. Studies on turning walking in post-stroke patients reported that the insufficient BF activation was associated with reduced knee flexion [11]. These studies revealed that multiple muscles, e.g. the RF, GS and BF, could be associated with the stiff-knee gait in post-stroke patients. However, little is known about how these muscles collectively work for knee flexion in post-stroke gait.

The normal and abnormal muscle activation can be assessed using the surface electromyogram (sEMG). The amplitude of sEMG reflects the recruitment and discharge rates of the active motor units and serves as an index of neuromuscular function. The higher sEMG amplitude indicates a more intensive muscle contraction. The spectral frequency of sEMG correlates with average conduction velocity and has been used to evaluate type I and II muscle fibers activation and the recruitment of motor units during muscle contraction [12]. Altered amplitude and frequency characteristics of sEMG signals has been observed in RF, BF and GS of post-stroke patients. For example, Boudarham et al found that the botulinum toxin injection in the RF could reduce the amplitude and spectral frequency of sEMG and could improve the knee flexion in the hemiparesis gait following stroke [13]. Li and Hong found when stroke patients took negative-heeled shoes during walking the sEMG amplitudes of GS and BF increased, accompanied by greater knee flexion [14]. Other studies reported that it was the GS but not the BF that exhibited reduced sEMG amplitude and frequency with decreased knee motion in post-stroke gait [15, 16]. These studies inspired us that to further examine the amplitude and frequency information underlying the sEMG signals of the involved muscles would help better understand the causes and solutions of stiff-knee gait in post-stroke patients.

The purpose of this study was to investigate the relationship between lower-limb muscle activation and the knee flexion during gait in the post-stroke hemiparesis patients. Effects of stroke were assessed on knee flexion and amplitude and frequency parameters of sEMG signals from the RF, GS, and BF contractions during walking. We hypothesized that the post-stroke patients would exhibit higher amplitude and frequency parameters in RF, but lower values in GS and BF on their paretic side than the non-paretic side, as well as than the healthy subjects. We also hypothesized that reduced knee flexion would be associated with simplified inter-muscular coordination in the post-stroke gait.

## Methods

### Subjects

Ten stroke patients (4 females and 6 males, age: 49.6 ± 12.9 y) and 10 gender- and age-matched (50.0 ± 13.2 y) healthy subjects participated in the experiment. The sample size was determined by a power analysis. The subject characteristics are shown in Table 1. The patients were recruited from the Department of Physical Medicine and Rehabilitation, Qilu Hospital, Shandong University from July 15th to July 27th, 2015. All the patients had been clinically diagnosed with first-ever stroke that occurred within 8 months. Both the patients and healthy subjects were able to walk at least 10 m independently with normal or correct-to-normal vision. The individuals with history of musculoskeletal injuries on their lower extremity, cervical and lumbar vertebra disorders, cardiovascular diseases, or tumor, cognitive difficulty and severe malnutrition were excluded. All subjects gave their written informed consent following the protocols approved by the Institutional Review Board of Shandong University after receiving detailed explanation of the purposes and potential risks of the experiment.

**Table 1:** Characteristics of stroke and healthy subjects.

Number	Stroke					Control
	Sex	Age (y)	Paretic side	Time post-stroke (months)	MBI*	Age (y)
1	M	49	R	8	95	51
2	F	31	R	3	55	31
3	M	26	L	4	40	23
4	F	56	L	2	70	55
5	M	57	R	2	90	57
6	M	42	L	1	70	44
7	M	52	R	2	45	55
8	F	65	L	6	60	64
9	F	69	L	2	65	68
10	M	49	R	2	70	52
**Mean**		49.6		3.2	66	50.0
**SD**		12.9		2.1	16.6	13.2

* Modified Barthel Index, a measure of activities of daily living.

### Experimental set-up

**Fig 1:**
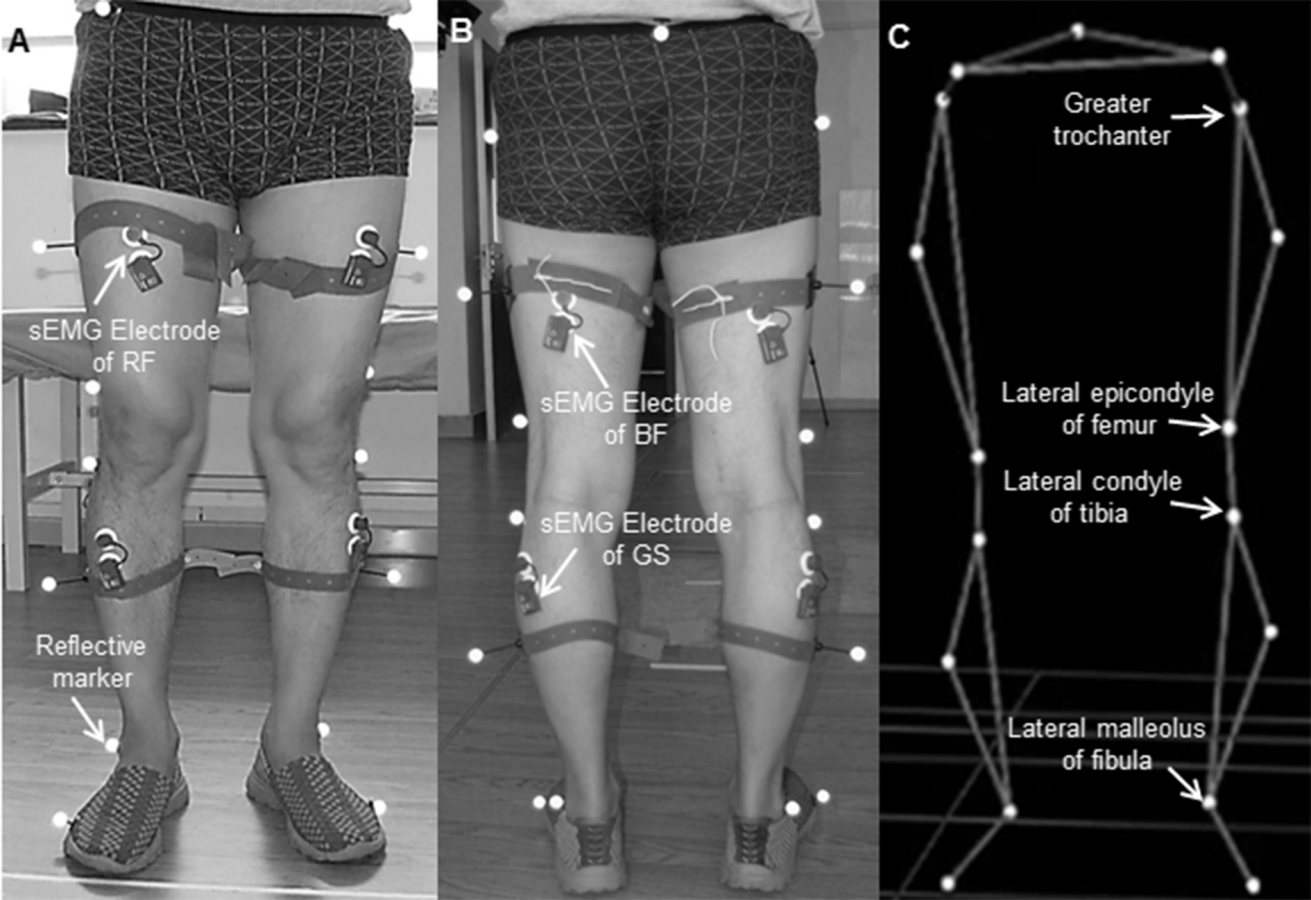
Positions of the retro-reflective marker sets and surface electromyography (sEMG) electrodes. (A) In a front view; (B) in a back view; (C) reconstructed kinematic model based on the marker sets.

A three-dimensional (3-D) motion capture system (BTS Bioengineering Corp, Italy) was used to obtain the gait kinematics. Six cameras measured the 3-D positions of 17 retro-reflective markers. The markers were affixed to the surface of the lower-extremity following the protocol of Davis R.B et al. (Fig 1C) [17]. Muscle activation during walking was synchronously recorded using a wireless sEMG system (BTS Bioengineering Corp, Italy) (Fig 1A and 1B). The electrodes of the RF were positioned at 50% on the line from the anterior spina iliaca superior to the superior part of the patella (Fig 1A). The electrodes of the BF long head were positioned at the midpoint of the line between the ischial tuberosity and the lateral epicondyle of the tibia (Fig 1B). Electrodes of the lateral GSs were attached at 1/3 of the line between the head of the fibula and the heel (Fig 1B). The kinematics signals were recorded at a sampling frequency of 200 Hz, and the sEMG signals were recorded at 1000 Hz. All relevant data are within the paper and its Supporting Information files (S1 Dataset and S1 File).

### Test protocol

All subjects were asked to walk a 3-m distance at a comfortable speed with their own shoes. Before walking the subject stood at a starting line waiting for command. After hearing the command, the subject walked straightly towards the finishing line. Each subject performed 10 trials with 1-minute rest between trials. Each subject was allowed to practice once or twice to be familiarized with the testing protocol.

### Data analysis

**Fig 2:**
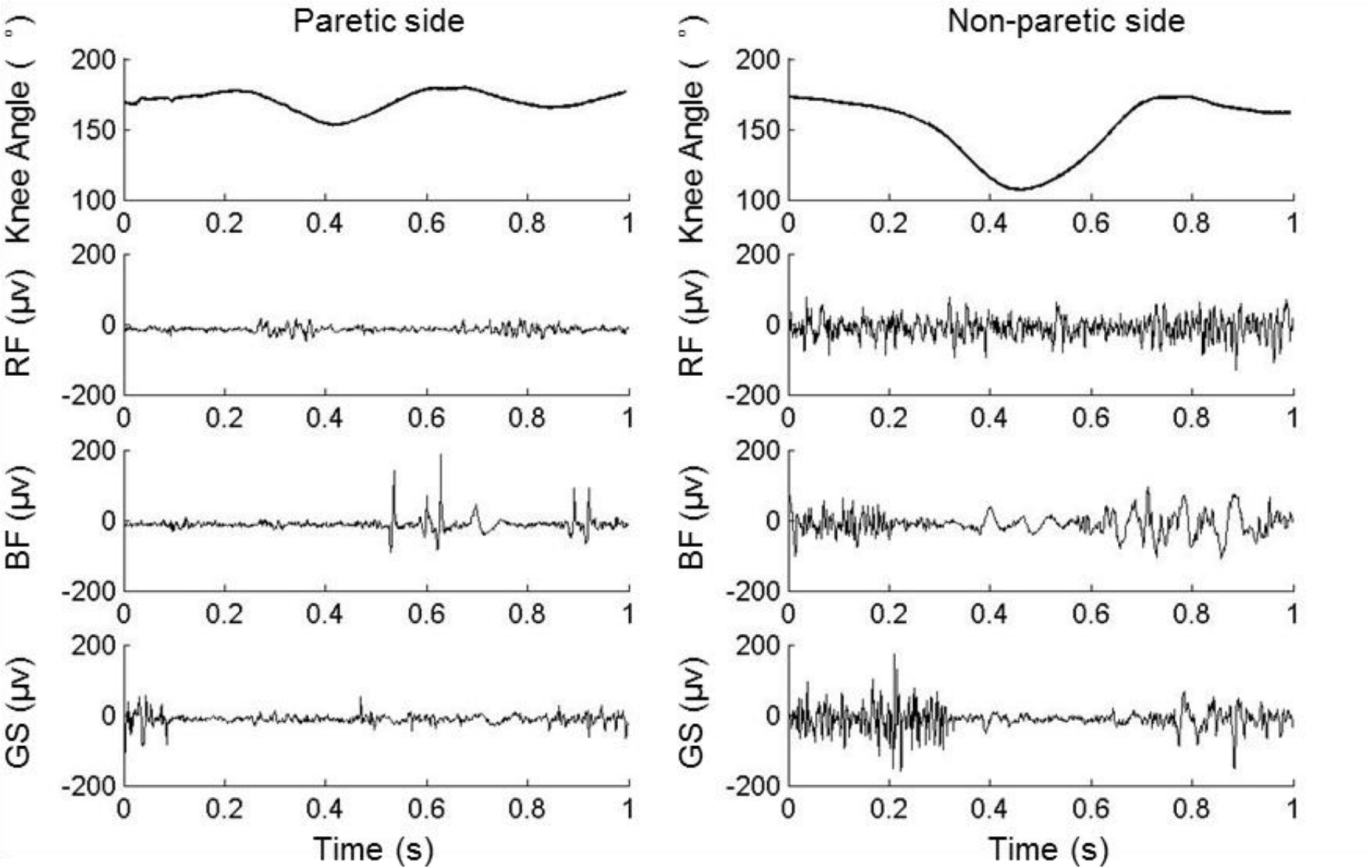
The knee joint angle and the sEMG signals of rectus femoris (RF), gastrocnemius (GS), and biceps femoris (BF) from a representative subject following stroke during one gait cycle.

**Fig 3:**
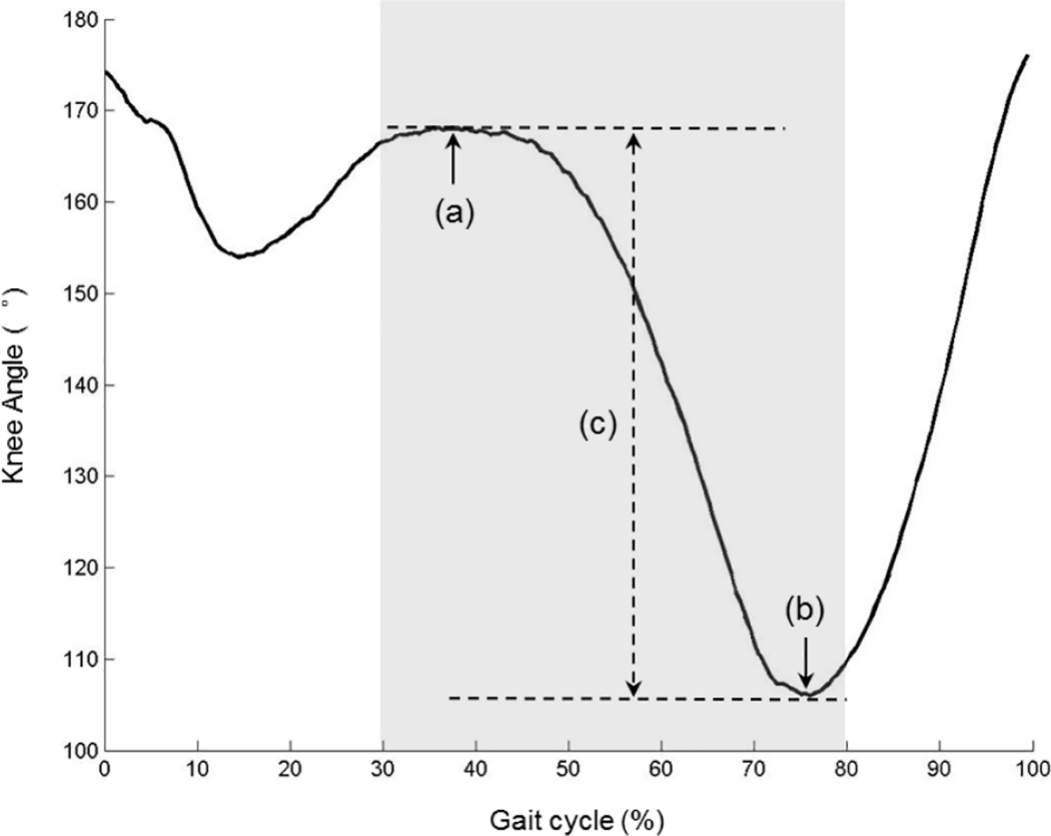
Definition of angle range (AR) of knee flexion. (a) The maximum of knee angle; (b) the minimum of knee angle; (c) the AR that was defined as the range between the maximum and the minimum of knee angles. The shaded part represents the duration from terminal stance (TSt) and initial swing (ISw) phases of a gait cycle.

The knee joint angles and the sEMG signals from the paretic side of a representative stroke patient are depicted in Fig 2. The knee joint angle in this study was defined as the angle formed by the line passing through the greater trochanter and the lateral epicondyle of femur, and the line passing through the lateral condyle of tibia and the lateral malleolus of fibula (Fig 1C). The angle range (AR) of knee flexion was the difference between the maximum and the minimum knee joint angle between the terminal stance (TSt) phase and initial swing (ISw) phase of a gait cycle (Fig 3). The gait trials with shielded markers were excluded, leaving 5 trials each subject for the following analysis. The bandpass width of sEMG signal was 10 Hz– 450 Hz, the decibel was 50 dB and the common mode rejection ratio was less than 750 mV RMS. The sEMG signals were not normalized to the peak sEMG amplitude during the gait [18]. The paretic and non-paretic sides of the healthy subjects are determined in accordance with their paired patient. For example, if one patient has left-side paralysis, then for the paired healthy subject the paretic side is the left and the non-paretic side is the right, and vice versa.

The amplitude of sEMG signals of the RF, BF and GS was quantified using the root mean square (RMS). The RMS is calculated as:

$$RMS=\sqrt{\frac{1}{n}\sum_{i=1}^{n}e{\left(i\right)}^{2}}$$ (1)

where the *e*(*i*) is the amplitude of the sEMG signal at each time point *i*, and the *n* presents the number of sampling points (i.e. the product of sampling frequency and time length) of sEMG signals.

The sEMG frequency characteristics were estimated using mean frequency (MNF), which is defined as:

$$f_{mean}=\frac{\int_{0}^{f_{s}/2}fP(f)df}{\int_{0}^{f_{s}/2}P(f)df}$$ (2)

where the *f*_*s*_ is the sampling frequency of the sEMG signal, and the *P(f)* is the power spectral density of the sEMG signal. The MNF is the mean frequency value of the sEMG spectrum. Short-Time Fourier Transform (STFT) was used to compute the time-frequency spectrum based on which the MNF was calculated. Considering the instability of sEMG signals, a shifting-window technique was used in the STFT, with the window width 30 ms and the shifts as long as 15 ms. The parameters were calculated using MATLAB 2014a (The Mathworks, Natick, MA, USA).

Statistical analyses were performed using SPSS 20.0 (SPSS Inc., Chicago, IL). Data were compared across the following three groups: (1) the paretic and (2) non-paretic sides of stroke patients, and (3) the controls (only the paretic side was selected). An independent *t*-test was applied to examine the differences between the stroke patients and controls. A paired *t*-test was used to examine the difference between the paretic and non-paretic sides of the stroke patients. No interaction was considered across these *t*-tests. Correlation analyses were performed between the sEMG parameters (RMS and MNF) of RF, GS and BF and the AR of knee flexion, respectively, on the paretic and non-paretic sides of patients and the controls. A stepwise multiple linear regression analysis was applied to find the associations between the AR of knee flexion and the RMS and MNF of RF, GS and BF for the paretic, non-paretic and control groups. A *p*-value of less than 0.05 was considered statistically significant.

## Results

**Fig 4:**
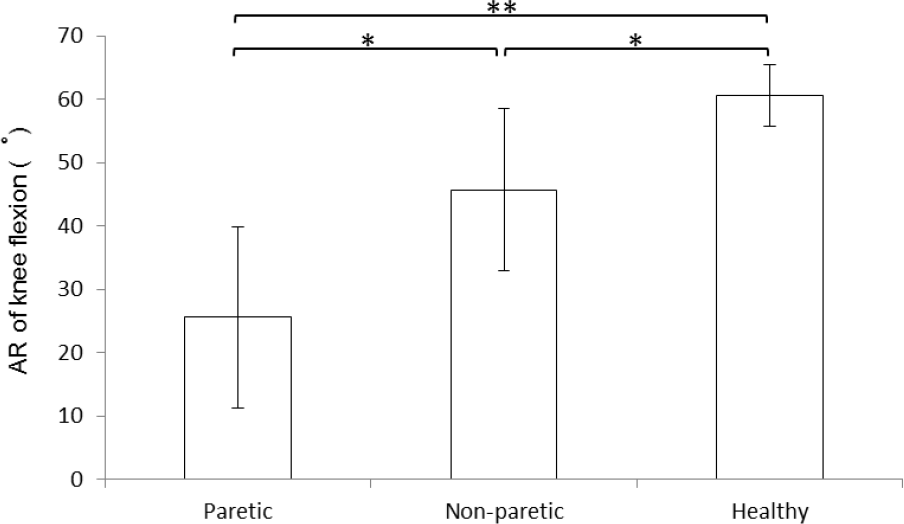
The AR of knee flexion on paretic and non-paretic sides and healthy subjects. * *p* < 0.05; ** *p* < 0.001.

Statistical analysis showed that the stroke significantly affected the knee flexion of gait (Fig 4). The AR of knee flexion were 27.58° ± 14.30° and 45.72° ± 12.78° on the paretic and non-paretic sides, respectively, for the stroke patients; and 60.57° ± 4.82° for the controls (Fig 4). Significant differences were observed between the paretic and non-paretic sides (*t* = 4.098, *p* < 0.01), between the paretic side and the controls (*t* = 7.331, *p* < 0.001), and between the non-paretic side and controls (*t* = 3.439, *p* < 0.01, Fig 4).

**Fig 5:**
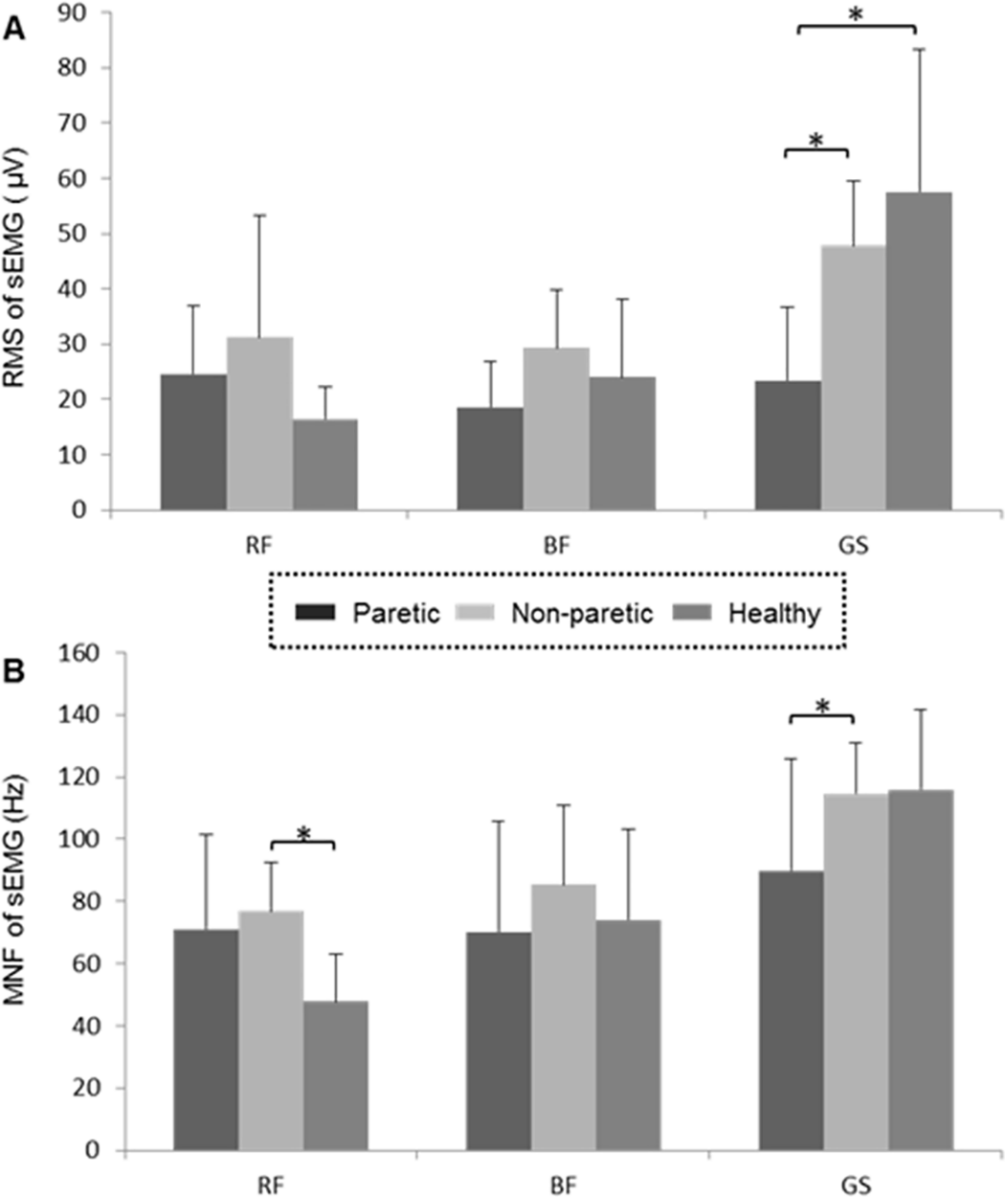
Muscle activities of the paretic, non-paretic and control groups. (A) Root mean square (RMS) of sEMG of RF, BF and GS; (B) mean frequency (MNF) of RF, BF and GAS. * *p* < 0.05.

Results of the RF, GS and BF contraction magnitudes during knee flexion are shown in Fig 5A. The GS of the paretic side showed significantly lower RMS than that of the non-paretic side (*t* = 3.635, *p* < 0.01) and the controls (*t* = 3.738, *p* < 0.01) (Fig 5A). No significant difference was observed in the RMS values of GS between the non-paretic side and the controls (*p* = 0.296, Fig 5A). Neither the RF nor the BF showed significant difference in RMS between the paretic and non-paretic sides (RF: *p* = 0.339; BF: *p* = 0.064), between the paretic side and the controls (RF: *p* = 0.087; BF: *p* = 0.314), or between the non-paretic side and the controls (RF: *p* = 0.067; BF: *p* = 0.357) (Fig 5A).

Results of MNF for the RF, BF and GS are shown in Fig 5B. The MNF of RF on non-paretic side was significantly higher than that of the controls (*t* = 2.495, *p* < 0.001, Fig 5B). No significant difference was found in MNF between the paretic and non-paretic RFs (*p* = 0.640) or between the paretic RF and that of the controls (*p* = 0.053) (Fig 5B). The GS of the paretic side showed significantly lower MNF values than that of the non-paretic side (*t* = 2.495, *p* < 0.05, Fig 5B). No significant difference was found in MNF of GS between the paretic side and the controls (*p* = 0.081) or between the non-paretic side and the controls (*p* = 0.883) (Fig 5B). Results showed that the stroke did not affect the MNF of BF (Fig 5B). No significant difference was found in the MNF of BF among the paretic, non-paretic and control groups (paretic vs. non-paretic: *p* = 0.313, paretic vs. control: *p* = 0.804, non-paretic vs. control: *p* = 0.65) (Fig 5B).

**Fig 6:**
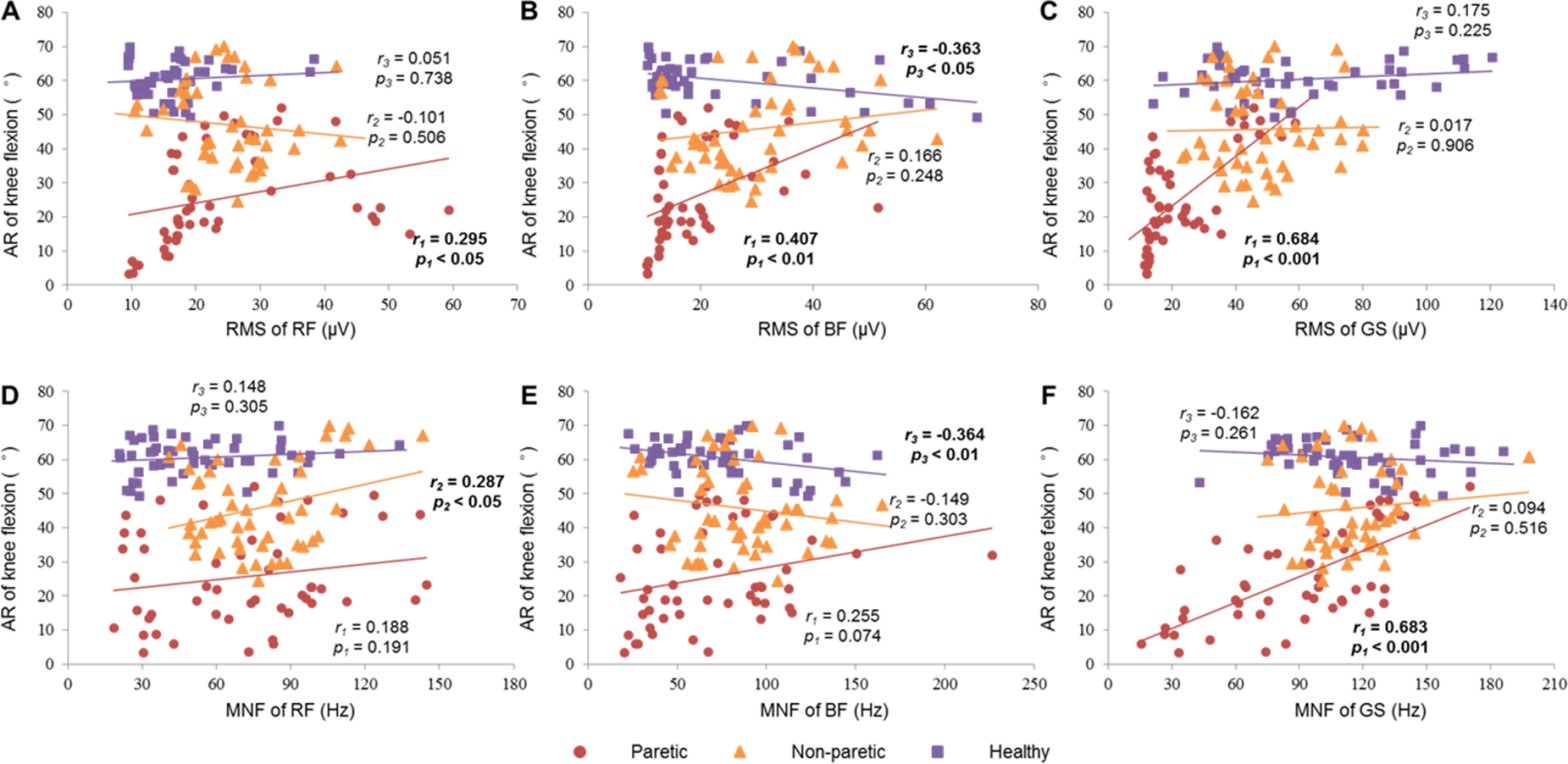
Correlations between muscle activation and angle range (AR) during knee flexion. (A)-(C): Correlations between the RMSs of RF (A), BF (B), GS (C) and AR. (D)-(F): Correlations between the MNFs of RF (D), BF (E), GS (F) and AR. The *r*1, *r*2 and *r*3 are the correlation coefficients of the paretic, non-paretic and control groups, respectively. The *p*1, *p*2 and *p*3 indicate the significance of the paretic, non-paretic and control groups, respectively.

The Fig 6 exhibits correlations between the muscle activities and the AR of knee flexion on the paretic, non-paretic and control groups. The AR was positively correlated with the RMSs of RF (*r* = 0.295, *p* < 0.05, Fig 6A), BF (*r* = 0.407, *p* < 0.01, Fig 6B) and GS (*r* = 0.684, *p* < 0.001, Fig 6C) on the paretic side of the patients. Neither for the non-paretic group nor for the control group had positive correlations between the RMSs of the muscles and the AR (Fig 6A, 6B and 6C). For the BF of healthy subjects, the RMS (*r* = -0.363, *p* < 0.05, Fig 6B) and MNF (*r* = -0.364, *p* < 0.01, Fig 6E) of BF showed negative correlations with the AR. No similar correlation was observed between the RMSs of BF and AR (Fig 6B), or between the MNF of BF and AR (Fig 6E) in stroke patients. Both the RMS (*r* = 0.684, *p* < 0.001, Fig 6C) and MNF (*r* = 0.683, *p* < 0.001, Fig 6F) of paretic GS were positively correlated with the AR. By contrast, no significant correlation was found between the RMS of GS and AR (Fig 6C), or between the MNF of GS and AR (Fig 6F) in either the non-paretic or the control group. The AR was correlated with the MNF of RF (*r* = 0.287, *p* < 0.05, Fig 6D) on the non-paretic side of patients. However, no significant correlation was found between the MNF of RF and the AR in either the paretic or the control group (Fig 6D).

Results of regression analysis are shown in Table 2. For both the non-paretic group and the controls, all the sEMG amplitude and frequency parameters, including the RMS and MNF of RF, BF and GS, were selected as predictive variables to the AR of knee flexion. Totally 65.6% and 45.2% variations of AR could be predicted by the variables for the non-paretic group and the controls, respectively. By contrast, for the paretic group only three sEMG parameters—the RMS of BF, the MNF of BF and the MNF of GS—had been selected by the stepwise regression as predictive variables to the AR. The three variables could explain 82.1% variations of AR for the paretic group (Table 2).

**Table 2:** Regression analysis of the angle range (AR) of knee flexion and muscle activities.

AR		Muscle activities							
		Constant	RF_RMS_	BF_RMS_	GS_RMS_	RF_MNF_	BF_MNF_	GS_MNF_	*r*^*2*^
**Paretic**	Coefficients	**-11.286**	**-**	**1.872**	**-**	**-**	**-0.350**	**0.296**	**0.821**
	Equation	AR = -11.286+1.872×BF_RMS_-0.35×BF_MNF_+0.296×GS_MNF_							
**Non-paretic**	Coefficients	**69.102**	**-0.565**	**-0.339**	**0.699**	**0.403**	**-0.311**	**-0.294**	**0.656**
	Equation	AR = 69.102–0.565×RF_RMS_-0.339×BF_RMS_+0.699×GS_RMS_+0.403×RF_MNF_-0.311×BF_MNF_-0.294×GS_MNF_							
**Healthy**	Coefficients	**118.292**	**-0.214**	**0.498**	**-0.193**	**-0.022**	**-0.371**	**-0.223**	**0.452**
	Equation	AR = 118.292–0.214×RF_RMS_+0.498×BF_RMS_-0.193×GS_RMS_-0.022×RF_MNF_-0.371×BF_MNF_-0.223×GS_MNF_							

## Discussion

This study investigated the knee flexion and the amplitude and frequency features of the RF, BF and GS activations and the multiple lower-limb-muscle coordination during the stance-to-swing phases of the gait cycle, by comparing the performance of stroke patients with that of the gender- and age- matched healthy individuals. The effects of stroke on the AR of knee flexion and on the RMS and MNF of RF, BF and GS between the TSt and ISw phases of gait were examined. Correlation analyses were used to identify specific lower-limb muscle activation with the range of motion of knee during the stance-to-swing phases of gait. The associations between the RMS and MNF parameters of the muscles and the AR of knee flexion were tested using a stepwise multiple regression analysis. Results demonstrated that stroke lowered the knee flexion of the paretic and even the non-paretic sides in comparison of the healthy subjects (Fig 4). The stroke possibly affected the contraction of GS, considering the GS of paretic side showed significantly lower RMS and MNF values than that of the non-paretic side (Fig 5). In addition, results showed that fewer muscle parameters could explain more variations of AR for the paretic group compared to the non-paretic group and the controls, suggesting a simplified muscle coordination following stroke (Table 2). These findings confirmed our hypothesis that it would be the multiple muscles’ coordination rather than single muscle contraction that should be responsible for the confined knee flexion of post-stroke gait.

The observation that knee flexion declined on the paretic side was consistent with previous findings from straight and turning gait of hemiplegic individuals [5]. The reduced knee flexion observed in this study was related not only to the ISw stage, but also to the TSt stage, highlighting the role of the terminal stance phase in knee kinematic control (Fig 4). The reduction of propulsion and knee flexion velocity at toe-off on paretic limb could result in the decreased knee flexion during swing [19, 20]. In addition, this study found that not only the paretic but also the non-paretic limb showed impaired knee flexion. The insufficient knee flexion on the non-paretic side could be attributed to the exaggerated propulsion during pre-swing and shortened swing time to compensate for the weakened and torpid paretic limb [21]. The difference in knee flexion between two limbs could contribute to the asymmetric gait of stroke patients, which may further increase the deflection of the center of gravity and increase the risk of falling during walking [22].

Despite a number of studies demonstrated that the confined knee flexion in post-stroke gait was due to the over-activation of RF [7, 8], different opinion about the role of RF in post-stroke stiff knee exists [9, 23]. In our study, we did not find any effects of stroke on the RMS of RF though the knee had shown significantly reduced knee flexion (Fig 5A). Instead, our study found an increase in the non-paretic MNF of RF compared to the controls (Fig 5B). These findings suggested that the declined AR of knee flexion on paretic side in hemiplegia gait was not confined by excessive activity of RF. Patients would reduce swing time of the non-paretic side to avoid the single support of the paretic side, which led to the knee flexion insufficient. Therefore, the RF on non-paretic side recruited more muscle units in the TSt and the ISw phases to increase forward acceleration, implying a potential compensatory mechanism that maintains the functioning of paretic limb by increasing the firing rate of the non-paretic muscles [13].

Results of the RMS and MNF also showed that the stroke affected the magnitude and frequency of the sEMG signals for the GS, but not for the BF (Fig 5). Stroke led to decreased magnitude and frequency of GS, revealing atrophy of muscle fibers, lowered firing rate, weakened capacity of motor-units recruitment, reduced conduct velocity and muscle fatigue by hemiplegia for gait performance [15, 24]. The stroke-induced neuromuscular abnormity in GS may result in less than necessary propulsion at the pre-swing phase and thus generate debilitated muscle force for knee flexion during swing stage, all of which could impair the AR of knee flexion in gait [25].

The RMS of RF, BF and GS was positively correlated with the AR of knee flexion only on the paretic sides of the patients (Fig 6A, 6B and 6C), revealing that the confined knee flexion in hemiplegia gait is associated with decreased magnitudes of lower-limb muscle contractions [11]. The magnitude and frequency parameters of GS were positively correlated with the AR on the paretic side (Fig 6C and 6F), which implies that the post-stroke stiff knee could largely depend upon the abnormal neuromuscular control of GS [25]. Both the RMS and MNF of BF showed significant correlations with the AR in controls rather than in patients (Fig 6B and 6E). This result suggests that knee flexion in healthy individuals was highly relied on the BF activation, while without the association between the BF and AR may indicate pathological changes of post-stroke gait.

By regression analysis this study further examined the relationship between the magnitude and frequency parameters of multiple muscles and knee flexion in post-stroke gait (Table 2). Although a few studies have established associations between lower-limb muscles’ coordination patterns and knee joint kinematics for healthy individuals [26, 27], less is known about how the stroke interferes with these associations during the stance-to-swing phases of the gait cycle. The changes in muscles’ activation patterns and muscle coordination during walking has been considered to give rise to the decreased knee angles [5]. The current study showed that, for both the non-paretic group and the controls, the RMS and MNF of all muscles, including the RF, BF and GS, were selected as predictive variables to the AR of knee flexion. For the paretic limb, however, only the RMS of BF and the MNF of BF and GS, were selected as variables in prediction of knee flexion during gait. This finding suggested that the knee flexion in post-stroke gait was dependent on fewer muscles’ activations, which is consistent with the previous finding that reduced muscle co-contraction on the paretic side would appear in hemiplegia gait [28]. It is noteworthy that the parameters of BF have selected as variables in the equations of paretic, non-paretic and control groups, implying the functioning of BF would be equally important for both the post-stroke and normal gait. This could also interpret why no significant difference was found in BF parameters across the three groups (Fig 5). Moreover, the regressive equations for the paretic group, the non-paretic group and the controls have different coefficients and constants, and the selected magnitude and frequency variables could explain 82.1% to 45.2% of variations of the knee flexion. Compared to the stroke patients, the knee flexion in healthy subjects did not rely on any specific muscles and thus exhibited weak association with the muscular parameters. By contrast, on the paretic side, fewer muscles were responsible for variations of knee flexion. These results imply that the inconsistent muscle coordination may be a contributor to the different AR of knee flexion among the three groups, which enriched our knowledge about the effects of stroke on the lower-limb neuromuscular control for knee flexion, and may facilitate the clinical evaluation and treatment of the post-stroke gait.

This study investigated the relationship between the knee flexion and the muscle activations including both the magnitude and frequency parameters of the sEMG in lower limbs during the stance-to-swing phases of a gait cycle. The stiff knee on paretic side of hemiplegia patient was related to the altered magnitude and frequency characteristics of the BF and GS, as well as the simplified muscle coordination. This study has some limitations. First, the RF, BF and GS were selected as representative muscles because of their roles in knee flexion and their locations that can be easily accessed when positioning the sEMG electrodes. It would be of interest how the other muscles surrounding knee, such as the vastus medialis or semitendinosus, contribute to the stiff knee of post-stroke gait. Second, this study applied simple and effective algorithms to analyze the signal and find out the pathological biomarkers of muscle contractions after stroke. To achieve more characteristics underlying the non-stationary sEMG signals, more powerful time and frequency analytical tools, such as the wavelet or empirical mode decomposition analyses are worthy to be applied. Third, the parameters entered in the regression analysis were not substantial. To select more effective parameters and to establish more suitable empirical equations, a larger number of parameters should be identified and enter the regression analysis.

## Conclusions

Stroke led to decreased knee flexion for both the paretic and non-paretic limbs and impaired the magnitude and frequency characteristics of GS and RF activation in gait performance. The knee flexion of paretic limb relied on fewer muscles, suggesting that the simplified muscle coordination would be a contributor to the compromised knee flexion in post-stroke gait. This study has implications for rehabilitation of stiff-knee gait in post-stroke hemiparesis patients.

## Supporting information

S1 Dataset Study database. (ZIP)

S1 File Supplementary information for the study database. (DOCX)

## References

[1] Wonsetler EC, Bowden MG. A systematic review of mechanisms of gait speed change post-stroke. Part 1: spatiotemporal parameters and asymmetry ratios. Topics in stroke rehabilitation. 2017:1–12. 10.1080/10749357.2017.1285746 PMC5875693 28220715

[2] Perry J, Garrett M, Gronley JK, Mulroy SJ. Classification of walking handicap in the stroke population. Stroke; a journal of cerebral circulation. 1995;26(6):982–9. 10.1161/01.str.26.6.982 7762050

[3] Wonsetler EC, Bowden MG. A systematic review of mechanisms of gait speed change post-stroke. Part 2: exercise capacity, muscle activation, kinetics, and kinematics. Topics in stroke rehabilitation. 2017;24(5):394–403. doi: 10.1080/10749357.2017.1282413 28218021 10.1080/10749357.2017.1282413 PMC5702549

[4] Boudarham J, Roche N, Pradon D, Delouf E, Bensmail D, Zory R. Effects of quadriceps muscle fatigue on stiff-knee gait in patients with hemiparesis. Plos One. 2014;9(4). 10.1371/journal.pone.0094138 PMC3981762 24718087

[5] Jung H, Ko C, Kim JS, Lee B, Lim D. Alterations of relative muscle contribution induced by hemiplegia: Straight and turning gaits. International Journal of Precision Engineering and Manufacturing. 2015;16(10):2219–27.

[6] Ostadal M, Chomiak J, Dungl P, Adamec O. Distal rectus femoris tendon transfer in cerebral palsy patients. Acta Chirurgiae Orthopaedicae Et Traumatologiae Cechoslovaca. 2007;74(6):388–91. 18198088

[7] Cruz AI Jr., Ounpuu S, DeLuca PA. Distal rectus femoris intramuscular lengthening for the correction of stiff-knee gait in children with cerebral palsy. Journal of Pediatric Orthopaedics. 2011;31(5):541–7. doi: 10.1097/BPO.0b013e31821f818d 21654463 10.1097/BPO.0b013e31821f818d

[8] Drefus LC, Buckland MA, Backus SI, Root L. The functional effect of a distal rectus femoris tenotomy in adults with cerebral palsy. Gait & Posture. 2014;40(1):145–9. 24742707 10.1016/j.gaitpost.2014.03.017

[9] Knuppe AE, Bishop NA, Clark AJ, Alderink GJ, Barr KM, Miller AL. Prolonged swing phase rectus femoris activity is not associated with stiff-knee gait in children with cerebral palsy: A retrospective study of 407 limbs. Gait & Posture. 2013;37(3):345–8. 22959561 10.1016/j.gaitpost.2012.07.034

[10] Francis CA, Lenz AL, Lenhart RL, Thelen DG. The modulation of forward propulsion, vertical support, and center of pressure by the plantarflexors during human walking. Gait & Posture. 2013;38(4):993–7. 23787149 10.1016/j.gaitpost.2013.05.009 PMC3795949

[11] Chen IH, Yang YR, Cheng SJ, Chan RC, Wang RY. Neuromuscular and biomechanical strategies of turning in ambulatory individuals post-stroke. Chinese Journal of Physiology. 2014;57(3):128–36. doi: 10.4077/CJP.2014.BAC204 24826781 10.4077/CJP.2014.BAC204

[12] Farina D, Merletti R, Enoka RM. The extraction of neural strategies from the surface EMG. Journal of Applied Physiology. 2004;96(4):1486–95. doi: 10.1152/japplphysiol.01070.2003 15016793 10.1152/japplphysiol.01070.2003

[13] Boudarham J, Hameau S, Pradon D, Bensmail D, Roche N, Zory R. Changes in electromyographic activity after botulinum toxin injection of the rectus femoris in patients with hemiparesis walking with a stiff-knee gait. Journal of Electromyography and Kinesiology. 2013;23(5):1036–43. doi: 10.1016/j.jelekin.2013.07.002 23928281 10.1016/j.jelekin.2013.07.002

[14] Li JM, Hong YL. Kinematic and electromyographic analysis of the trunk and lower limbs during walking in negative-heeled shoes. Journal of the American Podiatric Medical Association. 2007;97(6):447–56. 18024839 10.7547/0970447

[15] Alcan V, Canal MR, Zinnuroglu M. Using fuzzy logic for diagnosis and classification of spasticity. Turkish Journal of Medical Sciences. 2017;47(1):148–60. doi: 10.3906/sag-1512-65 28263483 10.3906/sag-1512-65

[16] Hill E, Housh T, Smith C, Schmidt R, Johnson G. Muscle- and Mode-Specific Responses of the Forearm Flexors to Fatiguing, Concentric Muscle Actions. Sports. 2016;4(4). 10.3390/sports4040047 PMC5968893 29910296

[17] Davis RB III, Ounpuu S, Tyburski D, Gage JR. A gait analysis data collection and reduction technique. Human Movement Science. 1991;10(5):575–87.

[18] Lamontagne A, Richards CL, Malouin F. Coactivation during gait as an adaptive behavior after stroke. Journal of Electromyography and Kinesiology. 2000;10(6):407–15. 11102843 10.1016/s1050-6411(00)00028-6

[19] Burpee JL, Lewek MD. Biomechanical gait characteristics of naturally occurring unsuccessful foot clearance during swing in individuals with chronic stroke. Clin Biomech (Bristol, Avon). 2015;30(10):1102–7. 10.1016/j.clinbiomech.2015.08.018 26371855

[20] Anderson FC, Goldberg SR, Pandy MG, Delp SL. Contributions of muscle forces and toe-off kinematics to peak knee flexion during the swing phase of normal gait: an induced position analysis. Journal of Biomechanics. 2004;37(5):731–7. doi: 10.1016/j.jbiomech.2003.09.018 15047002 10.1016/j.jbiomech.2003.09.018

[21] Allen JL, Kautz SA, Neptune RR. Forward propulsion asymmetry is indicative of changes in plantarflexor coordination during walking in individuals with post-stroke hemiparesis. Clinical Biomechanics. 2014;29(7):780–6. doi: 10.1016/j.clinbiomech.2014.06.001 24973825 10.1016/j.clinbiomech.2014.06.001 PMC4157942

[22] Lewek MD, Bradley CE, Wutzke CJ, Zinder SM. The relationship between spatiotemporal gait asymmetry and balance in individuals with chronic stroke. Journal of Applied Biomechanics. 2014;30(1):31–6. doi: 10.1123/jab.2012-0208 23677889 10.1123/jab.2012-0208

[23] George-Reichley DG, Higginson JS. Potential Muscle Function During the Swing Phase of Stroke Gait. Journal of Applied Biomechanics. 2010;26(2):180–7. 20498489 10.1123/jab.26.2.180

[24] Nadiv Y, Vachbroit R, Gefen A, Elad D, Zaretsky U, Moran D, et al Evaluation of Fatigue of Respiratory and Lower Limb Muscles During Prolonged Aerobic Exercise. Journal of Applied Biomechanics. 2012;28(2):139–47. 22723112 10.1123/jab.28.2.139

[25] Sarre G, Lepers R. Neuromuscular function during prolonged pedalling exercise at different cadences. Acta Physiologica Scandinavica. 2005;185(4):321–8. doi: 10.1111/j.1365-201X.2005.01490.x 16266373 10.1111/j.1365-201X.2005.01490.x

[26] Lo J, Lo O-Y, Olson EA, Habtemariam D, Iloputaife I, Gagnon MM, et al Functional implications of muscle co-contraction during gait in advanced age. Gait & Posture. 2017;53:110–4. 28129590 10.1016/j.gaitpost.2017.01.010 PMC5346031

[27] Tennant LM, Maly MR, Callaghan JP, Acker SM. Analysis of muscle activation patterns during transitions into and out of high knee flexion postures. Journal of Electromyography and Kinesiology. 2014;24(5):711–7. doi: 10.1016/j.jelekin.2014.06.011 25127491 10.1016/j.jelekin.2014.06.011

[28] Gray VL, Pollock CL, Wakeling JM, Ivanova TD, Garland SJ. Patterns of muscle coordination during stepping responses post-stroke. Journal of Electromyography and Kinesiology. 2015;25(6):959–65. doi: 10.1016/j.jelekin.2015.09.003 26475243 10.1016/j.jelekin.2015.09.003

