# Supplementary material for: Associations between lower-limb muscle activation and knee flexion in post-stroke individuals: A study on the stance-to-swing phases of gait
Source: PLoS One. 2017 Sep 8;12(9):e0183865. doi: 10.1371/journal.pone.0183865 (PMC5590852; doi:10.1371/journal.pone.0183865)
Supplement: S1 File — (DOCX) [file pone.0183865.s002.docx]

**Supplementary information for the study database**

The dataset of this study have been uploaded in the system with a file name “S1_Dataset.zip”. After decompression, there are totally two folders, one for the healthy group and the other one for the patients. In each group, there are ten sub-folders named ‘1’, ‘2’, ‘3’, ‘4’, ‘5’, ‘6’, ‘7’, ‘8’, ‘9’, ‘10’. Each sub-folder includes the raw data of one subject. In each sub-folder, there are five more sub-folders named ‘1’, ‘2’, ‘3’, ‘4’, ‘5’, including the surface electromyography (sEMG) signals (saved as ‘emg.csv’) and the kinematic signals (saved as ‘point.csv’) of the first to the fifth trial. To fully de-identify the subjects, the subject number was randomized, so that the subjects’ numbers in the database do not correspond to those exhibited in Table 1.

In each ‘emg.csv’ file, the ‘EMG signal 1’, ‘EMG signal 2’, ‘EMG signal 3’ and ‘EMG signal 4’ represent the EMG signals of the rectus femoris, biceps femoris, tibialis anterior and gastrocnemius of the right limb, respectively. The ‘EMG signal 5’, ‘EMG signal 6’, ‘EMG signal 7’ and ‘EMG signal 8’ represent the EMG signals of the rectus femoris, biceps femoris, tibialis anterior and gastrocnemius of the left limb, respectively. The sEMG signals were sampled at 1000 Hz and were recorded in μV.

A ‘point.csv’ includes three-dimensional coordinates of 17 marker points attached upon the lower limbs. The ‘r thigh.X’, ‘r thigh.Y’, ‘r thigh.Z’ are the coordinates of the right greater trochanter; the ‘r knee 1.X’, ‘r knee 1.Y’, ‘r knee 1.Z’ are the coordinates of the right lateral epicondyle of femur; the ‘r knee 2.X’, ‘r knee 2.Y’, ‘r knee 2.Z’ are the coordinates of the right lateral condyle of tibia; and the ‘r mall.X’, ‘r mall.Y’, ‘r mall.Z’ are the coordinates of the right lateral malleolus of fibula. For the left side, signals have the similar nomination. The kinematic data were sampled at 200 Hz and were recorded in meter.
